# Family relationships as a source of narrative identity of people with advanced dementia

**DOI:** 10.1186/s12877-023-04258-6

**Published:** 2023-09-08

**Authors:** Urszula Kłosińska, Magdalena Leszko

**Affiliations:** 1 Faculty of Psychology, SWPS University of Social Sciences and Humanities, ul. Ostrowskiego 30B, Wrocław, 53-238 Poland; 2 Department of Psychology, University of Szczecin, Szczecin, Poland

**Keywords:** Advanced dementia, Narrative identity, Family, Critical discourse analysis

## Abstract

**Background:**

The growing body of research on narrative identity, while helpful, rarely focuses on people with dementia. In this paper, we explore how individuals living with advanced dementia construct their narrative identities in relation to their family experiences, which play a crucial role in shaping identity as shown by recent studies.

**Methods:**

We conducted a qualitative study using data from 15 semi-structured interviews with people aged 66 to 94 who have advanced dementia. The data were analyzed using a textual-oriented discourse analysis.

**Results:**

We identified two discourses—autobiographical and economic—that organize their narrative identities. Through the autobiographical discourse, participants emphasized their sense of belonging within a social group and their role as custodians of family identity. Within the economic discourse, they negotiated their social utility and value, particularly in response to demeaning discourses targeting individuals who do not accumulate wealth. In the structural analysis, we identified two narrative types—looped or unfolding—that depend on their affective experiences related to their family. We especially explored how the repetition of narrative threads by individuals with dementia might indicate a traumatic background rather than just memory disruptions.

**Conclusions:**

This study provides insights into the narrative identities of individuals with advanced dementia, shedding light on the intersection of family experiences and identity formation in this population.

## Background

Narrative identity is ‘a person’s internalized and evolving life story, integrating the reconstructed past and imagined future to provide life with some degree of unity and purpose’ [1]. Considered an important aspect of personality, narrative identity persists inasmuch as our ability to accumulate new experiences. Through expressing self-defining stories we reveal who we are, explain how and why we become particular people, communicate what matters most in our lives, give meaning to our experiences, and maintain a sense of self-continuity [2].

Illness disrupts life continuity which provides new circumstances for meaning-making and structuring our self-stories [3]. Narratively framing ourselves and our illnesses affects our quality of life, mental health, and potential for recovery [4]. Integrating the meaning of stressful experiences also impacts our ability to cope with stress and can slow or accelerate biological aging [5]. Therefore, recently the function of narrative identities has been studied in the context of living with chronic somatic diseases, such as endometriosis [6], HIV [7] or cancer [8], and coping with various types of mental disorders like mood disorders [9], bipolar disorder [10], psychosis spectrum [11], PTSD [12], and borderline personality [13]. To the best of our knowledge, the narrative identities of people with dementia have been studied far too rarely [14–18]. It is interesting as dementia is widely considered one of the biggest challenges of the 21st century due to the increasing number of its cases and incurable status which causes significant public health burden to individuals, families, and society [19]. As the population ages, research that deepens our knowledge about dementia’s impact on narrative identity is warranted.

There are at least two reasons why studying the narrative identity of people living with dementia is not common. First, narrative identity has usually been operationalized through the efficacy of autobiographical memory [20]. The two most common symptoms of dementia are impairment of memory and a gradual loss of communication abilities [21]. Thus, people with dementia, whose autobiographical memories are disturbed, were considered as doomed to lose their sense of self [22]. However, our previous study [23] showed that forasmuch as people with dementia can communicate with others, they create meaningful and complex narrative identities. They do so by organizing their narrative identity discursively even in advanced stages of dementia, despite severe memory and language impairment. Second, people with dementia are often stigmatized as unable to participate in research procedures [24]. As McAdams listed [25], there are several approaches to examining narrative identity. Some authors use standardized methods such as *Life-story Interview* [26], *Awareness of Narrative Identity Questionnaire* [27], or analysis of written personal narratives in response to a given prompt [28]. Others encourage participants to freely construct narrations and then researchers code their responses for objectively defined constructs such as: agency and communion themes, redemption and contamination, elaborative processing, meaning-making, and coherence [29]. Finally, participants themselves are asked to perform self-ratings on their descriptions [30, 31]. Because of aphasia and memory impairment, people with advanced dementia could have difficulty with self-rating, writing a longer self-story, or keeping an imposed topic in mind. Thus, traditional methods of measurement may not be effective in exploring their narrative identities. In this study we examined the narrative identities of people with dementia by implementing discourse analysis. This method allows us to extrapolate meaningful data from a range of fragmentary material of varied granularity. Our aim was to search for identity-related textual markers even in enunciations of people unable to freely construct narrations suitable for Adler’s methodology or with speech impairments requiring constant interviewer’s engagement, without which no longer narratives could be constructed and recorded.

We aim to investigate how family experiences influence the narrative identity of individuals with dementia. It is well established that family, as the primary social group, plays a crucial role in developing identity [32]. However, most research focuses on adolescents and it is unclear how family relationships shape the narrative identity of individuals with dementia. Researchers stressed that family roles can have a potential impact on the identity of individuals with dementia [33–35]. People with dementia often have limited social networks with family members being the only meaningful units [36]. This study builds on existing literature by broadening the perspective of individuals with dementia. Therefore, we explore how people with advanced dementia discursively constructed their narrative identities in relation to family experiences. Our study focuses on two research questions:

1. How do people with advanced dementia discursively construct their narrative identities?
2. What discourses do they use to express their family relationships?

## Materials and methods

### Theoretical framework

The concept of narrative identity has been studied using empirical approaches such as social constructivism, social psychology, cognitive narratology, and critical linguistic approaches [37]. In this study, we used a textual-oriented approach to discourse analysis (TODA) [38] to examine the narrative identity of individuals with late-onset dementia. This approach posits that identity is a fluid, fragmentary, contingent, and discursively constituted construct of selfhood rather than a static and finished entity [37]. It is shaped by language and social practices rather than external factors alone, such as material possessions, socioeconomic class, gender, or age [39]. This approach views identity as being embedded in a dynamic self-narrative and narrative analysis as a method of studying it [40]. Finally, it acknowledges that identities are positioned in relation to societal schemes and institutions of power [39] and, therefore, cannot be fully understood without considering the discourses that shape them. By discourse, we understand a set of practices of representing extralinguistic reality and a way of constructing social structures via language [41].

### Participants and study procedure

The data for the present study were part of a larger project, running from 2019 to 2021, examining the self-narratives constructed by people with advanced dementia in relation to discourses on old age, disability, and dementia. Analyses based on data from this larger study have been previously published [23]; however, the study reported there involves exploring the narrative identities of individuals with advanced dementia formed through their professional experiences. Fifteen people, chosen purposely from a group of 27 project participants, participated in this study. The eligibility requirements included: (1) a medical diagnosis of late-onset dementia, (2) presenting in the moderate to the severe stage (3) aged above 65, (4) retired, (5) retaining the ability to communicate, and who (6) constructed their narrative identity solely in relation to family experiences. None of the participants dropped out or refused to participate during the study.

The participants were aged from 66 to 94 (M = 81, SD = 7.71) and identified as women, with the exception of one man. They were diagnosed with dementia in the moderate to severe stages due to: Alzheimer’s disease (n = 7), vascular dementia (n = 3), dementia with Lewy bodies (n = 2), dementia due to mixed etiology (n = 2), and progressive supranuclear palsy (n = 1). All of them needed assistance in daily activities. Twelve of them had lived in the long-term care (LTC) facility for at least five months (M = 18, SD = 7.52), and three people who lived in their own homes were supported by their relatives. Only 2 women were married and the other participants were widowed (n = 12) or divorced (n = 1). All of them have children with whom they have regular contact. Most of the participants had secondary education, but the sample also included people with primary (n = 2), vocational (n = 1), or higher education (n = 3). We summarized the study sample characteristics in Table 1.

**Table 1:** Study participants

Initials	Sex	Age	Diagnosis	Education	Marital status	Children	Place of residence (in months)
DC	F	69	PSP	Higher	Divorced	1	LTC (5)
DM	F	87	AD	Secondary	Widowed	3	LTC (23)
BR	F	94	VD	Secondary	Widowed	4	LTC (23)
TP	F	83	MIXED	Secondary	Widowed	1	LTC (24)
MC	F	82	AD	Secondary	Widowed	3	LTC (12)
CB	M	79	DLB	Vocational	Widowed	1	LTC (15)
GK	F	90	DLB	Primary	Widowed	2	LTC (33)
JG	F	89	AD	Higher	Widowed	2	LTC (21)
JZ	F	75	MIXED	Secondary	Widowed	2	LTC (10)
KS	F	78	VD	Higher	Widowed	2	LTC (15)
KL	F	83	AD	Secondary	Widowed	1	LTC (21)
EP	F	88	VD	Secondary	Widowed	1	LTC (10)
DN	F	80	AD	Secondary	Married	1	Private home
BB	F	66	AD	Secondary	Married	1	Private home
JD	F	73	AD	Primary	Widowed	1	Private home

M: male, F: female, LTC: long-term care facility, AD: Alzheimer’s disease, DLB: Dementia with Lewy bodies, VD: Vascular dementia, MIXED: Dementia due to mixed etiology, PSP: Progressive supranuclear palsy

Individuals with dementia belong to a vulnerable population and their comfort is of prime importance. The study protocol was approved by the Ethical Review Board at the SWPS University of Social Sciences and Humanities in Poland and while conducting the study, we analyzed ethical guidelines [42–44]. The participants were recruited from geriatric and neuropsychology clinics and two long-term care (LTC) facilities in Poland. We invited them face-to-face to take part in conversational-style interviews that were pilot tested in a group of five people in 2019. All interviewees provided both written and verbal consent to participate after obtaining approval from their relatives and LTC managers. The interviews were semi-structured and focused on exploring the self-image of people with dementia, their relationships, and their world, in the context of their cognitive impairment, functional disability, and aging (see Table 2). This interview structure was used through the entire project.

**Table 2:** Semi-structured interview

Research field	Main question
Selfhood Relationships Surroundings Aging Ability Dementia	Can you tell me something about yourself? What people are important to you? Can you tell me about the place you currently live at? What is ‘aging’ to you? How do you understand ‘ability’? What is ‘dementia’ to you?

All participants were encouraged to answer our questions and, depending on their responses, we continued our inquiries to broaden the scope of their stories. All interviews were conducted one-on-one by the first author in private rooms at the participants’ current living facilities. The first author also made field notes containing interpretative comments, body language, facial expressions, feelings, and the content during each interview. One interview was conducted with each participant without repetition. Interviews lasted from 48 to 79 min (M = 59; SD = 9.49). Interviews were voice-recorded and transcribed using a simplified Jefferson transcription system [45]. The transcripts were not returned to the study participants.

### Data analysis

The authors started their analysis by coding the dataset to focus only on those fragments in which the participants created self-narrations. Transcripts were coded by the first author with the use of the MaxQDA software (v.20.4.1). The units of coding that an independent qualitative researcher reviewed included: self-perception, relationships with loved ones (both past and present), family experiences (positive and negative), self-events (occurring during childhood, adulthood, and old age), and the individual’s image of the future.

Using TODA, we conducted a two-step iterative textual analysis of each case, including linguistic analysis (focusing on lexico-grammatical aspects) and interdiscursive analysis (examining genres, discourses, and textual styles). Following Halliday [46], we assumed that lexico-grammatical forms of language are the primary carriers of meaning and are used unconsciously to reflect external reality. Therefore, when analyzing discourses, we were interested in both the semantic and syntactic aspects of the utterances, their functions within the situational context, and the social practices they accomplished. To compare our findings, we looked for common linguistic, discursive, and textual patterns among all cases in the dataset. Finally, we contextualized our results within the broader socio-historical context to understand the ideology underlying the identified discourses.

Here, we redacted any information that could identify our participants. The quotes were translated from Polish to English and proofread for accuracy. In order to preserve the lexical and syntactic aspects of the original utterances, along with any errors in spelling, logic, punctuation, and grammar that may have occurred due to aphasia, their translations are intentionally literal, even if this results in somewhat “disjointed” English.

We applied various strategies to strengthen the trustworthiness of our analysis [47]. To increase credibility and consistency, we used investigator triangulation, peer examination of our findings, audit trails, and reflexivity on our own biases. We also reached data saturation with eight interviews, beyond which no new relevant findings emerged from the dataset. To enhance the transferability of our findings, we provided a rich description of the context, research procedure, and study participants. We also aimed to maximize diversity in the study sample to allow for a greater range of applications for the research findings.

## Results

All participants in this study produced complex narrations about themselves despite major cognitive difficulties, such as anomia or severe autobiographical memory impairment. In response to the first question: *Can you tell me something about yourself?*, individuals began describing their inner worlds, including complex descriptions of settings, unexpected events, psychological/physical responses, unplanned actions, attempts, and consequences, as outlined in Herman [48]. Their narrations convey the experiences of living through their storyworld, evaluations of themselves and others, and interpretations of how various events shape their current self. We divided the results into two parts reflecting the structure of our analysis. The first part presents the results of a content analysis of the interviewee narratives, examining how their autobiographical and economic discourses convey their affective relationship with their families. The second part focuses on narrative structure to show how it connects with positive or negative evaluations of family experiences.

### Involvement in the relationship with the family members

Past and present relationships with family members were recurrent themes that organized the narrative identities of all participants in this study. Twelve people described themselves by reconstructing their family story. We identified this as a marker of autobiographical discourse [cf. 49]. However, three interlocutors focused on their current health problems rather than on telling their family stories. They stressed how their current situation made them financially dependent on their children. In this section, we will show how those two groups construct meaningful narrative identities by using autobiographical or economical discourses.

### Autobiographical discourses

Twelve out of fifteen study participants created their narrative identities in relation to their family history. Their narratives are dominated by autobiographical discourse. We distinguished a set of grammatical markers of this discourse. First, our interlocutors often use first person plural forms (‘we’) denoting ‘me and my family’ to describe their relationships with their parents, siblings, spouse, or children. By association with ‘we’, they explicitly profile themselves as a part of a social group thus expressing belonging to a community [cf. 50]. In their stories, family members are indispensable life companions. They express that belonging to a family is an important aspect of their identity, even if their loved ones passed away or stopped living together (they moved to an LTC facility).

Participants whose narratives were dominated by the autobiographical discourse also organized the history of their family (and thus their own life story) spatially and not temporally. They could indicate important locations of life events in the family context, but they could not precisely pinpoint the time of those events. We illustrate this with the following two extracts:

/DN, age 80, AD/

DN: Because I am a potato (.)

I: A potato?

DN: Potato (.) [*laughing*]

I: So you come from Greater Poland Voivodeship?

DN: Yes. […] I was born right **there**. I lived **there** with my parents for some time. **There** too my father comes/ came from Włoszczkowa. It’s over **there** behind Rawicz too, right? So we are all potatoes.

I: And how did you find yourself here?

DN: Well, because my father got a job **here**, right? Well, he was the commander of the industrial guard. He was a military man by origin (.) Well, and **here** he got a job right? Well, and **this** is where we later moved to. (.)

I: And how old were you when you moved here to Silesia?=

DN: = Jesus. Gosh how old was I? (.) I already went to school because later I moved **here**. (.) You know what? Well, it’s hard for me to say how old I was, oh gosh.

/CB, age 79, DLB/

CB: I: had/ y: have two sisters. (.) My dad also. And i-i/ in the military we’ve been everywhere. In Siemianowice. In Łabędy. I drove also when I to the military jo-joi/ came (.) so **that** too y: y: in Gliwice I was. I had driven i-i-in Nysa. And in Nysa **there** is the centra:l/ this y:/ thereof **this**/ oh what is it called? (.) **This** intelligence is (.) central. **Here** at **this** old station. Now I’ve heard how they moved it to/ to the new station/ to **this** old station in Nysa. **There** were such: (.) fortifications. It’s **there** only grandma used to go. [*showing*] She always stayed like **that** and went in. Always everyday because her husband is buried **there**. And she told us that **there** i/ in-in-in (.) some year soldiers still crawle/ were coming out in fifties sometime. Solders (.) who didn’t know that the war was over.

I: Did I understand that you come from Nysa?

CB: No: not Nysa. I don’t. In Nysa I served.

I: So, where do you come from?

CB: From Siemianowice. I’m Silesian-born.

Both interlocutors begin their narrations by geographically contextualizing self-events by listing the names of places where these events occurred. By doing so, they also attribute regional identity to their family and themselves. DN introduces herself as *a potato* which is a regionalism used by people from middle Poland voivodeship to stress their rural origin and farmer heritage. CB introduces himself as *a Silesian-born*, identifying himself with another regional Polish identity. By emphasizing their regional identity they express communal belonging despite relocation to different regions or an LTC facility. DN also stressed the shared, familial aspect of regional identity by saying: *We are all potatoes*. Interestingly, our interlocutors manifested their regional origins from the beginning of our interviews. Utilizing the positioning theory assumption [51], we could state that they try to avoid being treated as an outcast, a person who is “*other* than *us*” [39]. Because being ‘other’ can be determined by geographical boundaries, the speakers stressed their regional origin to emphasize their self-perception as group members.

In the extracts below, besides using proper location names, DN and CB described places relevant to their narrative identity with spatial deixis, such as *here/there* and *this/that*. Therefore, they organized their narrative identities by evoking spaces, which remain meaningful and familiar to them despite having no clear reference for their interlocutors. Interestingly, even if DN and CB had difficulties with the temporal designation of life-story events, they still organized them, by switching between past and present tense to build causal connections between events, or referring to life stages associated with a particular activity (*I already went to school* and *when I to the military came*).

We noticed that our study participants used spatial references to denominate close relationships. They expressed that being physically close means being emotionally close to their loved ones. Those staying in their own homes indicated that their family members live nearby and mentioned places where they spent time together. For those who live in the LTC facility the primary attribute of emotional engagement was the frequency of physical visits by their loved ones. This is illustrated by a comment of one study participant:

/JZ, age 75, MIXED/

I was visited twice by my daughter and (.) and-a:nd my son too. They are very/very back me up. And I love them very much too.

Our participants often reconstructed their life stories in relation to their family history in a wider socio-historical context. In particular, World War II and living under a communist or Soviet regime were recalled mostly. See the extract below:

/BR, age 94, VD/

BR: We’ve had it tough because y: when the Ruskies entered, well then, my father had to flee from the east/ eastern areas of Volhynia.

I: And why did he have to flee Volhynia?

BR: Well it’s simply because y: dad married a Ukrainian woman. And ma’am y: Ruskies when they entered they just sim/ for sure/ anyway all y: friends who then lived in that eastern area of Volhynia, so Ruskies deported everyone. They shot everyone. They destroyed everyone! But father y he was interested in those situations their/ those people what Ru/ pe/ they lo/ Ruskies do what like after-after that. (.) and just from the news it came out that he would definitely not survive. So he left us in Volhynia alone. And alone he de: departed to the family in Siedlce.

BR narrates her tough childhood situation by referencing historical background of massacres of Poles in Volhynia and Eastern Galicia. She precisely identifies the cause of her situation – a cross-cultural marriage, she therefore recognizes the historical backdrop of socio-political tensions between Poles and Ukrainians. She operates with the past tense to organize sequences of events and to present the causal relationship between historical events, family circumstances, and her own traumatic experiences. She expresses the affective engagement by emphasizing the phrase *they destroyed everyone* and by using a discursive marker of contempt by referring to Russians as *Ruskies*. Furthermore, she uses the conditional mode in relation to the past to present arguments for her father’s decision to escape Volhynia. She not only tries to reconstruct the past but also speculates about her father’s motivations.

In this section, we showed how participants in this study construct their narrative identities using autobiographical discourse. We identified the following markers of the autobiographical discourse: association with the first person plural ‘we-the family’ to express a sense of belonging to a social group, the spatial organization of self-events, assuming a regional identity, expressing a sense of emotional intimacy in relationships, and finally describing their family history in a broader socio-historical context to give meaning to self-events.

### Economical discourses

As mentioned initially, three participants made their health problems a leading theme of their narrative identity, family relationships and self-perception. These three women introduced themselves as ill-stricken and focused on reconstructing the history of their illness. However, their acceptance for their condition varied. DC negotiated the possibility to live independently and meet her daily needs on her own despite being aware of neurocognitive symptoms such as falls, memory and speech impairment. KS believed in the possibility of recovery and emphasized the efforts she put into the rehabilitation of left-sided palsy. Only EP seemed to accept her dementia and transfer to the LTC facility as a consequence of the progressing disability.

All three women maintained close relationships only with their children. Having lost their husbands in early adulthood required them all to fulfill the role of the sole economic supporters of their families. All three narratively depicted the family as an economic unit whose purpose is to finance the needs of its members. See the following extract:

/KS, age 78, VD/

[My daughter] is jealous that a mother-in-law y: bought a house for my son and her daughter as a wedding gift. She thinks I bought it. She on/ when she was 18, she wanted an apartment, so I gave her my apartment, which I had been paying for some years. And I signed the apartment over to her. My son wanted a car, so I bought him a Cinquecento, and now she thinks I bought him the house. I say ‘Tell me, where did I get the money? There is no dad. Where was I supposed to earn so many thousands for a house like that?’. After all, it cost over a million. He has a villa not a house. And now she is looking for a boyfriend too to tease him that he too/ that she too has a boyfriend with a house like that.

Here, KS describes family conflict using specific economic lexica. She narrates using verbs related to economic discourse, such as: *bought*, *gave*, *paying*, *signed over*, *earn*, *cost*, and lexica referencing the state of possession (*my apartment; signed over to her; he has a villa*), exchange relationships (*wedding gift; bought him a Cinquecento*), asset valuation (*thousand*; *over a million; a villa not a house)*. First, she described the relationship with her children, identifying it in terms of meeting financial needs: *my son/daughter wanted it, so I bought it/gave it*. By narrating her actions in the first person singular (*I did*), she stressed her independence and self-efficacy in managing her property. This discourse allowed KS to give meaning to the emotions of her daughter and explain her negative affect (jealousy) as a manifestation of economic unfulfillment. She also describes motivations through economic discourse – her daughter seeks a partner to be able to economically rival her son’s fortune. Thus, she describes the motivation for having close relationships as being connected to the desire to multiply wealth. Finally, through economic discourse, she marks relationships of power and superiority. In her story, people constantly compete economically to gain social superiority. Her daughter competes with her son over who gets more from her and KS competes with the son’s mother-in-law, whose economic status allowed her to buy *a villa*, while KS can only afford to buy *a Cinquecento*, or *sign over an apartment*. She therefore narratively explains to herself why she cannot meet the needs of her daughter, who makes excessive demands of her.

The economic discourse was used by all three participants in the context of their illness to anticipate the needs of their family members, give meaning to their affects and motivations, and define power relationships. The extracts below illustrate these features:

/DC, age 69, PSP/

I: What is the most frightening thing about a disease-related old age?

DN: Well that we will be on o-ourselves/ o-on: on someone’s dependence. That it will be a burden. (…) [*continues in a crying voice*] Because it will be if/ the burden fo:r the family. And a huge one.

/KS, age 78, VD/

KS: My son pays for me.

I: And how do you take care of this place? About the common spaces here? Because the halls/=

KS: =Well, it’s a pity there aren’t any stores. There is completely nothing here. This is such a hole. My son sends me whatever I want. He is the one who sent me coffee. He sent me waffles. He always sends me fruits. So, he takes care of me.

/EP, age 88, VD/

EP: My husband left a lot of plots. Gradually my son is selling [it]. From that he pays me here. […] At the moment it is important for me this stay here. I told you I passed by my house and without a shrug.

I: And what would you like to achieve by being here?

EP: [*sights*] Mrs. Jane keeps asking me how long we will be here. And I say ‘as long as our children have enough money’. If they run out of money, they have to take us home. Because being here is not for free. The fees are high.

I: Mhm.

EP: It’s just we are. That’s why I say ‘You know Mrs. Jane as if we don’t have money, then we have things packed and to mine’.

Here, all three women emphasize dependence on their children’s decisions and financial capabilities. However, this dependency evokes different feelings for each of them. DC perceives her illness as a financial problem for the family. She uses an idiom to express fear of being *a huge burden* to her family. During the interview, she struggled with accepting this narrative identity and speculated it would have negative consequences on her daughter’s career and family life. This internal conflict manifested linguistically in unfinished sentences, stuttering, a crying voice, and the pronouns she used. She objectified her doubts, attributing them to a communal ‘we’ (*We will be*), or hiding her agency behind an unspecified entity (*It will be*) instead of taking them upon herself (*I am a burden*). In contrast, KS has consistently viewed familial relations as an exchange of economic goods. She openly admits that her son pays for her stay at the LTC facility and lists items he purchases to fulfill her needs. She concludes that this is proof of his devotion and care. EP described her son selling off his father’s assets to pay for her stay at the LTC facility. She stresses the need to stay at the LTC facility as long as possible. However, she is worried that this might not be possible for financial reasons. She expresses those concerns using plural pronouns (*We will be here*) and presents them as part of a dialogue with LTC staff members to objectify her worries, distance herself from them or find comfort.

In this section, we discussed how three participants frame their narrative identities within an economical discourse. We showed how they created the image of the family serving a mutual fulfillment of economic needs. We stressed that within economic discourse they defined power relationships and gave meaning to both their own and their loved ones’ affects and motivations.

### Positive and negative evaluation of familial experiences

Structure-wise, participants in this study created two types of narratives: unfolding or looped. The former was used by twelve interviewees whose families were a source of positive experiences and feelings of safety. They constructed their narrative identities by proceeding from one self-story to another. Through these stories they highlighted character traits and important needs, presenting multiple self-images. Their stories also had a clear temporal organization. By referring to the past, the interviewees sought to explain who they had become. By talking about the future, they expressed their needs and expectations. See the extract below:

/GK, age 90, DLB/

GK: My husband was a good (.) man. I: missed him very very much. And I miss him still, I say, because (.) because so many years and yet I can’t e: forget. […] Well, and I was left alone you know? And that’s why my grandchildren/ grandson ga:ve me here to be taken care of. Because he says/ they would/ they were afraid that:/ (…) They work. They go to work and I’m alone so I might not make it to the phone and/

I: And why do you need to be taken care of Mrs. G?

GK: Well because/ well because I’m alone. Well. And a/ and it’s/ my age I already have (.) ma’am. That’s why the grandchildren (.) were afraid that something would happen to me. That m: I might-might fall over.

Participants who described positive family experiences identified with shared family values. They used familial relationships with living and deceased loved ones to exemplify being helpful, caring, and capable. Interestingly, two women used the present tense to talk about their deceased relatives. Such behavior is usually interpreted as confabulation. Nevertheless, in this case, the relationships with deceased relatives were a source of self-efficacy and an internalized value system. Therefore, the use of the present tense here is not just a grammatical mistake, a symptom of memory disruption, but marks a discursive practice to retain the relationships that are crucial to the current self-perception. Let us illustrate this with an extract from our conversation with JG.

/JG, age 89, AD/

JG: And I had such a Grandmother. My beloved good Nanny. She was teaching me everything. […] All the times we went on trips. Sometimes we went to the mountains for a couple of days, so that there would be this movement.

I: With grandma?=.

JG: =Yes, yes. So n:-nice memories I have. She has fond memories. I have fond memories. We’re good together. Sometimes see. Meet each other. Talk. Sit. One to co:me to the other.

I: You meet your grandma?

JG: Of course, I also sometimes meet.

I: But when you’re 89 years old, how old is your grandma?

JG: [*laughs*] Grandma is holding up well.

I: Did I understand correctly that grandma is still alive?

JG: Yes. Grandma is from a family of (.) long-lived. (.) And she also knew how to do everything. She knew how to do everything.

[…]

I: Can you tell me who you are now?

JG: Everything and nothing. [*laughing*]

I: What does it mean?

JG: When I’m needed I’m taken here. When I’m needed somewhere else I’m taken somewhere else. Then they come to me and tell me: ‘Grandma let’s go here. Or let’s go there. Or let’s go this.’ And well, we go together. (.) I embroidered a lot of things. I crocheted. Because I, in turn, learned from my Grandma. And my Grandma always said: ‘Remember, learn everything so you know everything in life so no one surprises you’.

Here, JG described her grandmother as a life teacher whose guidance gave her a sense of being *needed*, enabling positive interactions with others. Although JG denied her grandmother’s death, she did so cheerfully by arguing that her grandmother *is holding up well* as she is of *the long-lived*. The interviewee operated with two temporal frames: past, when she described her grandmother’s actions (*was teaching*, *we went on trips, she knew how to do everything*), and present, to describe her continuous relationship with her grandmother (*we’re good together*, *meet*, *talk*). Thus, she separated the grandmother who was a life teacher (past) from the grandmother with whom she retains a bond (present). She used this discursive procedure to maintain a relationship with the deceased grandmother even if by contaminating both past and present agency. Moreover, using objectifying independent speech she referred to herself as *Grandma*, the same lexeme she used to address her grandmother. Thereby, she both identified with her grandmother’s values and interiorized this authority figure becoming a life teacher for the younger generations. Accepting the loss of the relationship with her grandmother would be to kill the *Grandma* identity within herself.

We identified the looped narration in three interviews. Its key feature was an iteration of the same story multiple times even when the conversation moved on to other topics. It seemed as if our interviewees had only one or two stories to tell about themselves. The lists of repeated threads were: (JD) living with an alcoholic husband + an abandonment by mother, (DM) being exiled with the entire family to a prisoner-of-war camp, (KL) mother’s death + abuse by father, brother, and husband. All these threads were clearly related to trauma and a loss of security. These looped narratives emphasized heroism or suffering, reinforcing a consistent narrative identity self-image of a hero (JD) or a victim (KL, DM). JD posed as a survivor that was able to live and raise her son against all circumstances. KL angrily repeated that her father, brother, and husband forced her to do exhausting physical work. She declared that she despised all men and used invectives and irony to express her rage, calling them: ‘loafer’, ‘great lord’, ‘dump’, or ‘sucker’. Instead of attempting to positively reevaluate her experiences, she relentlessly emphasized being the victim of the harmful male agency. DM described herself as a victim of war. In her narrative everything happened before, during, or after the war. She did not engage in any non-war topics, giving only yes or no answers to our inquiries. When she mentioned her war experiences, her phrases became longer and she shared her emotional attitude by increasing speech tempo or silencing her voice.

These women focused not on describing the traumatic self-event but on its impact on their current situation. We want to illustrate this with the example of JD. She repeated the story about abandonment by her mother five times during the interview. She explains this caused her to never befriend her sister. At the end of our conversation, she negotiated if and how the separation from her sister impacted her loneliness.

/JD, age 73, AD/

JD: Well you know, because I know that I supposedly have this family somewhere but I don’t have right? And I would be happy too right? If I had, right? [

I: [ Mhm.

JD: Toward oneself/ ones/ well, but that’s how it must have been, you know? And/ and that’s wh/ why not once I’m not what what/ not what as it should be, right.

I: And what you should be?

JD: Because! I feel so lo:nely. I know that somewhere out there in the world I have a sister and I don’t know her, right? I’ll tell you it’s also you know a bit overwhelming. Except that I’ve already learned to live with it, right? Now I say I’m alone, right?

Ultimately, participants in this study created two types of narratives: unfolding and looped. Our interlocutors predominantly used the former. For them, the family was a source of positive experiences and provided a sense of security. They integrated family values and described important traits for self-image through family relationships: helpfulness, caring, and self-reliance. As a result, they created complex narrative identities consisting of many self-images, consecutively uttered in new self-stories. In contrast, people who created looped narrations did not identify with their families. They iterated traumatic events related to family experiences. Within these stories they constantly returned to a single self-image of a hero or a victim. They were unable to move beyond this trauma-induced structure, to which they subordinated their entire narrative identities. Without voicing these traumatic stories, it would be impossible for them to express their identity.

## Discussion

Our study shows that despite significant memory loss and aphasia, people with advanced dementia construct complex and coherent self-stories which fulfill the narrative criteria [48]. Referencing social constructivism theory [52], we demonstrate that our interviewees narrated all three aspects of identity. They maintained a personal identity (Self 1) by using first-person pronouns such as “I,“ “me,“ “mine,“ “my,“ and “our.“ They described their personal attributes (Self 2) by talking about who they were, are, and will become in the future, as well as by describing their traits, skills, and beliefs. Those individuals also exhibited a social self (Self 3) by presenting themselves in conversations with us and describing how they are positioned in relationships with important others. Based on these results, we conclude that the belief that dementia itself leads to a loss of identity [22] is not supported by our findings.

Recent debates problematize whether people with dementia can generally participate in research [53, 54]. Therefore, we conducted semi-structured interviews in a casual conversational style at the participants place of residence to allow people with dementia to comfortably engage in self-narratives rather than conforming to the demands of the research procedure. We utilized TODA to focus on narrative identity-related textual markers in their utterances, rather than on cognitive deficits that impeded their understanding. This approach allowed us to interpret the data obtained without prior theoretical assumptions and thus “give voice” to those with dementia themselves [55]. We agree with Shannon, Montayre, and Neville [56] that research inclusivity is paramount in respecting the fundamental human rights of people with dementia to freedom of expression and participation in debates on health policies that concern their lives.

Our participants shaped their narrative identities by depicting themselves and their social roles in relation to their family experiences. Family researchers stress that every family creates its own identity, which is a set of its norms, power relations, events, and heritage (ethnicity, nationality, class, race) [32]. The family also produces its own consumption practices that shape its structure, values system, and forms of communication between its members [57]. These family aspects - social, historical, and economic were stressed by our participants who employed autobiographical and economical discourses in constructing their self-narrations.

The use of autobiographical discourse allows people with advanced dementia to emphasize belonging to a social group and functioning as the keepers of a family identity. Their narrative identities had a hierarchical structure, similar to the one described by Sabat and Harre [52] or Marsh and Shavelson [58]. The self (*I*) was a part of a family (*we*), which was a part of a regional group (*Silesian-born*, *the potato*) with shared historical memories (*Volhynia Massacre*). Participants organized their narratives spatially using spatial deixis to reference significant places and emphasize relations between physical and emotional proximity. As discussed by Chen [39], such discursive practices define who is inside or outside a social group, therefore our participants used these both to avoid exclusion and pose as storytellers passing knowledge to younger generations. That social role often conforms to social expectations towards older people [59].

Three participants defined family power relations with economic discourse, exhibiting neoliberal economic principles [60]. Within this framework, they positioned themselves as a burden to wealth generation, as women dependent on men, or as a former asset disposer who believes that her children owe her a debt of gratitude. Their descriptions presented a model in which the non-working subject becomes a social ‘parasite’ when not producing or multiplying value. This issue was discussed by Burch [61], who described disablist hate speech used to politically justify cutting financial support and vent feelings of frustration related to financial instability. Perhaps, that is why these three women negotiate whether their past agency and usefulness outweigh their current economic dependency to emphasize their social utility. Interestingly, these individuals internalized the economics of later adulthood, rather than the dominant socialist economic policies of their youth. This would imply that people with dementia can incorporate new ideological systems into their narrative identity, despite clear episodic memory disorders manifested by favoring past over present knowledge [cf. 62].

The last finding shows that people with dementia structure their narrative identity differently regarding the affective value of their family experiences. If family was a source of positive experiences and feelings of safety, they constructed unfolding narratives consisting of many self-stories, presented multiple self-images, and identified with family values. However, if they experienced family-related trauma, their narrative identity looped, becoming an iterated trauma story with a singular self-image of a hero or a victim. Vankeen et al. [12] show that narratives of traumatic experiences were less coherent, reflected unfinished attempts at meaning-making, and shared similarities with continuous rumination. Thus, perseveration of narrative threads by people with dementia is not just a symptom of cognitive impairment [63] but may also mark a non-adaptive form of coping with important life-events of a possible traumatic background.

### Limitations

Our study has several limitations. One is the dominance of women in the research sample. Women more often than men tend to respond to prompts asking for narratives, seem to focus on interpersonal domains, and share more developed narratives [64]. They also dominate in the population of people with late-onset dementia [65]. Future research should therefore expand to capture more male family-related narratives. Furthermore, considering the gender-age specificity of our interviewees, we recognize the potential need for additional discursive studies that concentrate on family economics and gender roles in Poland. These studies would be instrumental in providing a more comprehensive context for understanding the discourses of women with advanced dementia. This, in turn, is crucial for gaining insights into how social habitus influences their narrative identity. Another limitation is that the majority of participants were nursing home residents, whereas the majority of people with dementia in Poland live in their own homes [66]. Moving to a LTC can threaten the expression of identity for older adults with dementia due to institutional restrictions and limited social contacts outside of these facilities [67]. However, we did not find significant differences in the construction of narrative identities between people living at home and LTC residents. This may be due to the limited number of people living at home included in the study. Further research should examine this group of people in greater depth. Finally, in order to make the findings accessible internationally we inevitably lose some valuable interpretive leads in the translation process. Some dialect phrases or broken off syllables had to be omitted as we could not find an English equivalent for them. However, we have taken the greatest efforts to make the translation as close to the original as possible, preserving grammatical and semantic distortions and aphatic features in participants’ utterances.

## Data Availability

The data that support the findings of this study are available on request from the corresponding author, [UK]. The data are not publicly available due to their containing information that could compromise the privacy of research participants.
